# Acute White Matter Integrity Post-trauma and Prospective Posttraumatic Stress Disorder Symptoms

**DOI:** 10.3389/fnhum.2021.742198

**Published:** 2021-09-29

**Authors:** Carissa N. Weis, Ashley A. Huggins, Tara A. Miskovich, Jacklynn M. Fitzgerald, Kenneth P. Bennett, Jessica L. Krukowski, E. Kate Webb, Terri A. deRoon-Cassini, Christine L. Larson

**Affiliations:** ^1^Department of Psychology, University of Wisconsin-Milwaukee, Milwaukee, WI, United States; ^2^Sacramento VA Medical Center (VHA), Sacramento, CA, United States; ^3^Department of Psychology, Marquette University, Milwaukee, WI, United States; ^4^Montana VA Health Care System, Helena, MT, United States; ^5^Division of Trauma and Acute Care Surgery, Department of Surgery, Medical College of Wisconsin, Milwaukee, WI, United States

**Keywords:** PTSD, DTI, trauma, white matter, brain structure

## Abstract

**Background:** Little is known about what distinguishes those who are resilient after trauma from those at risk for developing posttraumatic stress disorder (PTSD). Previous work indicates white matter integrity may be a useful biomarker in predicting PTSD. Research has shown changes in the integrity of three white matter tracts—the cingulum bundle, corpus callosum (CC), and uncinate fasciculus (UNC)—in the aftermath of trauma relate to PTSD symptoms. However, few have examined the predictive utility of white matter integrity in the *acute* aftermath of trauma to predict *prospective* PTSD symptom severity in a mixed traumatic injury sample.

**Method:** Thus, the current study investigated acute brain structural integrity in 148 individuals being treated for traumatic injuries in the Emergency Department of a Level 1 trauma center. Participants underwent diffusion-weighted magnetic resonance imaging 2 weeks post-trauma and completed several self-report measures at 2-weeks (T1) and 6 months (T2), including the Clinician Administered PTSD Scale for DSM-V (CAPS-5), post-injury.

**Results:** Consistent with previous work, T1 lesser anterior cingulum fractional anisotropy (FA) was marginally related to greater T2 total PTSD symptoms. No other white matter tracts were related to PTSD symptoms.

**Conclusions:** Results demonstrate that in a traumatically injured sample with predominantly subclinical PTSD symptoms at T2, acute white matter integrity after trauma is not robustly related to the development of chronic PTSD symptoms. These findings suggest the timing of evaluating white matter integrity and PTSD is important as white matter differences may not be apparent in the acute period after injury.

## Introduction

In the United States, nearly 90% of people experience a traumatic event in their lives (Kilpatrick et al., 2013). While most individuals are resilient after trauma, a substantial subset of trauma-exposed individuals go on to develop posttraumatic stress disorder (PTSD) (Cloitre et al., 2019). PTSD is characterized by symptoms that include re-experiencing the traumatic event through intrusive thoughts, nightmares, and flashbacks, avoiding trauma-related stimuli, general hyperarousal, and experiencing negative thoughts or emotions that begin or worsen after the event (American Psychiatric Association, 2013).

Critically, at this point in time, clinicians do not have an accurate method of predicting who is at risk of developing PTSD after a traumatic event. Ideally, psychological and/or biological markers measured early after trauma exposure could be used to identify those at risk for chronic distress so that appropriate interventions could be administered (Bryant, 2003; Shalev et al., 2019). Localizing disruptions in the brain that underlie maladaptive behaviors and cognitions is important so that targeted interventions can be implemented to prevent symptom development without disrupting unrelated and intact neural processes.

Structural integrity studies using diffusion tensor imaging (DTI) have repeatedly yielded results suggesting the utility of white matter integrity as a biomarker of PTSD (Bremner, 2005; Karl et al., 2006; Alexander et al., 2007; Pitman et al., 2012). DTI is a neuroimaging technique that can non-invasively characterize structural integrity of white matter tracts in the brain by measuring the signal attenuation of water movement within brain tissue (Mori and Tournier, 2014). White matter tracts are the myelinated axons of neurons within the brain that facilitate the efficient neural transfer of information. Fractional anisotropy (FA) is an approximation of the spatial coherence of white matter tissue (Mori and Tournier, 2014). Prior research has shown that FA, a measure of disruption of white matter integrity, of the cingulum, corpus callosum, and uncinate fasciculus is repeatedly linked with PTSD symptom severity and diagnosis (Abe et al., 2006; Jackowski et al., 2008; Bierer et al., 2015; Fani et al., 2015; Kennis et al., 2015; Hu et al., 2016; Saar-Ashkenazy et al., 2016; Weis et al., 2018).

The cingulum interconnects fronto-limbic regions along the cingulate gyrus (Schmahmann et al., 2007), and the corpus callosum connects many prefrontal and parietal cortical regions between cerebral hemispheres (Hofer and Frahm, 2006). The structural connectivity of both the cingulum and corpus callosum has been hypothesized to underlie dysregulated emotions and memory processes (e.g., re-experiencing symptoms) present in PTSD (Mahan and Ressler, 2012; Pitman et al., 2012; Sanjuan et al., 2013; Reuveni et al., 2016; Akiki et al., 2017; Siehl et al., 2018). Finally, the uncinate fasciculus, a white matter tract which connects the temporal and frontal cortices through the limbic region, has also been implicated in aberrant extinction learning processes leading to avoidance behaviors in PTSD (Jovanovic and Ressler, 2010; Von Der Heide et al., 2013; Olson et al., 2015; Koch et al., 2017; Harnett et al., 2018).

Cross-sectional studies in a variety of trauma samples have shown that *lesser* integrity (e.g., lower FA) of the cingulum (Abe et al., 2006; Fani et al., 2012, 2015; Sanjuan et al., 2013; Bierer et al., 2015; Reuveni et al., 2016), corpus callosum (Jackowski et al., 2008; Li et al., 2016; Saar-Ashkenazy et al., 2016), and uncinate fasciculus (Hanson et al., 2015; Koch et al., 2017) are related to *greater* PTSD symptoms. Meta-analytic work also notes the integrity of these three white matter tracts in relation to PTSD (Daniels et al., 2013; Jenkins et al., 2016; Siehl et al., 2018; Ju et al., 2020).

Longitudinal research has yielded some insights into the structural changes that accompany PTSD, indicating that increased integrity over time of the cingulum (Zhang et al., 2012; Kennis et al., 2015), corpus callosum (Sun et al., 2015), and uncinate (Fani et al., 2017) are related to less severe PTSD symptoms. In one notable example, Kennis et al. (2015) demonstrated increased cingulum integrity (FA) 6–8 months after treatment for PTSD and up to 4 years later corresponded with concurrent improved PTSD symptoms.

However, relatively few studies (Fani et al., 2012; Sun et al., 2015; Hu et al., 2016; Li et al., 2016; Saar-Ashkenazy et al., 2016; Harnett et al., 2020) have investigated the predictive utility of acute white matter structural integrity in conferring risk for prospective PTSD, particularly in traumatically injured civilian samples. Notably, in a mixed-sample of acute trauma survivors, lower FA in the uncinate 1-month post-trauma was significantly related to greater posttraumatic anhedonia (PTA), 6 months post-trauma (Fani et al., 2017). Similarly, when compared to trauma-exposed controls, motor vehicle crash (MVC) victims had lesser integrity in the cingulum, corpus callosum, and uncinate 2 days after their accident that correlated with greater PTSD symptom severity 6-month later (Sun et al., 2015; Hu et al., 2016). Still, there are some limitations to these few prospective studies including, fairly small sample sizes (*n* < 62), homogenous trauma populations, and comparing diagnostic groups.

Therefore, the current study adds to this body of literature aiming to assess whether *acute* post-trauma white matter integrity relates to *prospective* PTSD symptom severity by examining a large and diverse traumatically injured sample. In keeping with the few studies of acute trauma survivors to date, we predicted that lesser integrity of the cingulum, corpus callosum, and uncinate after traumatic injury will be related to greater PTSD symptom severity 6 months later.

## Materials and Methods

### Participants

Nine-hundred sixty-nine individuals treated for traumatic injuries in the Emergency Department (ED) of a Level 1 Trauma Center in Southeastern Wisconsin were recruited directly from the ED or by phone following ED discharge for the *Imaging Study on Trauma and Resilience* (iSTAR study: Bird et al., 2021; Webb et al., 2021a, b; Weis et al., 2021a, b). After expressed interest in study participation the participant received a complete verbal overview of the study and were screened to ensure eligibility. Participants provided written informed consent, and all procedures were approved by the Medical College of Wisconsin Institutional Review Board. Though 969 individuals were recruited for study participation, only 524 met eligibility criteria of which 279 were discontinued or withdrawn before the first study (i.e., first visit unable to be scheduled, visit scheduled but participant no-showed, discontinued interest in study participation). Thus, 215 eligible participants were enrolled in the study.

Inclusion criteria include those who met criterion A of PTSD diagnosis as defined by the Diagnostic and Statistical Manual- 5th edition (DSM-V). Individuals then completed the Predicting PTSD Questionnaire (Rothbaum et al., 2014) to evaluate 5 risk factors to chronic PTSD development including evidence of prior trauma, current trauma severity, dissociation during current trauma, childhood trauma exposure, and family history of psychopathology (max score = 5; all 5 risk factors). Those with a minimum score of 3, indicating greater risk of developing chronic PTSD, were considered eligible. This inclusion criteria means our sample represents a subset of trauma-exposed individuals who are at elevated risk of PTSD development. In addition, individuals were eligible if they were between the ages of 18–60, English speaking, and able to schedule their first research visit within 30 days of the trauma.

Exclusion criteria included head injury more severe than a mild traumatic brain injury (score of less than 13 on the Glasgow Coma Scale; Sternbach, 2000; Teasdale et al., 2014), spinal cord injury with neurological deficit or any condition affecting brain structure or function, self-inflicted injury, severe vision or hearing impairments, history of psychotic or manic symptoms, currently on antipsychotic medications, clear substance abuse noted in medical record, on police hold following their injury, contraindications for MRI scanning including metal objects or fragments in the body, claustrophobia, and pregnancy or planned pregnancy within the next 6 months.

### Procedure

Participants came to research visits at two time points, within 2–4 weeks (T1; range = 3–33 days, mean = 18 days) and 6-months (T2; range = 157–231 days, mean = 187 days) following the trauma that resulted in their ED admission. At both visits, a large battery of behavioral, cognitive, self-report questionnaires, and neuroimaging data were collected. Herein, we report select measures from both time points and the structural and diffusion MRI (DTI) data from T1. As part of this larger study participants were scanned on two consecutive days within the acute post-trauma period (T1). Of the 215 enrolled participants, 208 were scanned in the MRI environment on Day 1, and 185 were scanned on Day 2 when the DTI protocol was completed. Of the 185 scanned, a total of *N* = 171 participants had complete diffusion weighted imaging scans. After visual inspection, four participants were removed for marked distortions in the DTI scans (*N* = 167). Of the 167 participants with usable DTI scans at T1, 148 had T2 PTSD data.

There were no differences in age, sex, or T1 (PCL-5) or T2 PTSD symptoms (assessed with Clinician Administered PTSD Scale; CAPS-5; see Section “Measures”) between individuals with usable scan data included in all subsequent analyses and those who were enrolled in the study but did not complete or have usable scans. Although, those enrolled in the study without scan data had significantly lower screened risk of PTSD as measured by the Predicting PTSD Questionnaire (Rothbaum et al., 2014) than those included in the current analysis, *t*(248) = −8.32, *p* < 0.001. See Table 1 for characteristics of the final sample (*N* = 148).

**TABLE 1:** Sample characteristics (*N* = 148).

	*M* (*SD*)
**Age**	33.12 (10.48)
**Sex**	65 male, 83 female
**Race**	**% of sample**
Asian	<5%
Black	60%
Pacific Islander	<5%
White	26%
More than one	6%
Unknown/not reported	6%
**Annual income**	**% of sample**
Less than $40,000	58%
40,000–60,000	16%
60,000–80,000	14%
Greater than $80,000	12%
**Educational attainment**	**% of sample**
Some high school	7%
High school graduate or GED	33%
Some college	28%
Associate degree	16%
Bachelor degree	14%
Master degree or higher	<5%
**Mechanism of injury**	**Percent (# male, # female)**
Assault	15% (8 M, 15 F)
Fall	1% (0 M, 2 F)
MVC	69% (47 M, 56 F)
Pedestrian struck	4% (4 M 2 F)
Other	11% (6 M, 8 F)
**Days since injury**	***M* (*SD*)**
T1	17.83 (5.79)
T2	183.64 (11.89)
**Prior trauma history (weighted LEC)**	31.39 (16.37)
**PTSD symptoms**	***M* (*SD*)**
PCL-5 (T1)	26.78 (17.29)
CAPS-5 severity (T2)	12.70 (11.25)
CAPS-5 Dx (T2)	27+/121−

*MVC, motor vehicle crash; LEC, life events checklist; PTSD, posttraumatic stress disorder; PCL-5, PTSD checklist for DSM-5; CAPS-5, Clinician Administered PTSD Scale for DSM-5; T1, 2-weeks post traumatic injury; T2, 6-months post traumatic injury; M, mean; SD, standard deviation; Dx, diagnosis. Small sample sizes for select racial groups are reported as <5% to ensure participant anonymity, thus cumulative percentages exceed 100%.*

### Measures

Prior trauma history is a significant risk factor in chronic trauma-related outcomes (Karam et al., 2014), therefore, to assess past traumatic/stressful experiences, we used the Life Events Checklist (LEC; Gray et al., 2004). The LEC assesses occurrence of 17 major life events (e.g., natural disaster, assault, combat, life-threatening illness, or injury) that a person may have experienced, witnessed, or learned about happening to someone close to them. To capture the greater PTSD risk conferred by experiencing an event as compared to learning about an event (Gray et al., 2004), a newly developed weighted summary score was used (Weis et al., 2021a). Items experienced firsthand were weighted by a factor of 3, items witnessed weighted with a factor of 2, and items learned about were weighted with a factor of 1. After weighting, all items were summed (maximum score = 102). Higher scores therefore indicate more events experienced with close proximity to the individual (descriptive information presented in Table 1).

#### Posttraumatic Stress Disorder Symptom Measurement

The Clinician-Administered PTSD Scale for DSM-5 (CAPS-5) was used to assess chronic PTSD symptoms at T2 (Weathers et al., 2013). The CAPS-5 is an interview consisting of 18 questions corresponding to PTSD criterion in the DSM-5. Frequency and intensity of PTSD symptoms are assessed by the interviewer and a single severity rating is designated for each item. Total symptom severity is derived from the sum of severity ratings on all questions. The interview was administered by trained senior study personnel and audio-recorded for each participant. A random selection of interviews (∼20%) were reviewed and reevaluated by licensed clinical psychologists in the study team to establish good reliability across interviewer administration within the study (Cohen’s Kappa = 0.95). At T2, the sample had predominantly subclinical symptoms (*M* = 12.70, *SD* = 11.25), as only 18% of participants (13 male and 14 female) met criteria for PTSD diagnosis according to the CAPS-5 (Weathers et al., 2013).

To assess baseline PTSD symptoms, we used total scores from the PTSD Checklist for DSM-5 (PCL-5) administered at T1 (Blevins et al., 2015; U.S. Department of Affairs, 2017). The PCL-5 is a 20-item self-report measure that assesses symptoms of PTSD according to the DSM-5 criteria. A sum score is generated reflecting symptom severity ranging from 0 to 80, with a higher scoring indicating greater symptom severity. Note, the PCL-5 was also administered at T2, however, the CAPS-5 is viewed as a more valid and objective measure of PTSD, therefore we used the CAPS-5 as the outcome variable of interest at T2 for all analyses rather than the PCL-5. Both measures of PTSD were highly correlated at T2, *r*^2^ = 0.44, *p* < 0.001.

#### Depression Symptom Measurement

Given the high degree of comorbidity of PTSD and depression after traumatic injury (Rytwinski et al., 2013; Flory and Yehuda, 2015), and in particular the relationship of depression and DTI after trauma (Isaac et al., 2015), we included depression symptoms as a covariate in the analysis. Depressive symptoms were assessed at T1 using the Depression, Anxiety and Stress Scale (DASS; Lovibond P.F. and Lovibond, 1995; Lovibond S.H. and Lovibond, 1995; Henry and Crawford, 2005). The DASS is a 21-item self-report measure divided into 3-subscales (7-items per subscale), depression, anxiety, and stress. Total scores from the depression subscale were used to evaluate depressive symptoms over the prior week relative to the time of administration. On the depression subscale total scores between 0 and 9 suggest asymptomatic depression, 10–13 indicate mild depression, 14–20 moderate depression, 21–27 severe depression, and scores greater than 28 correspond with extremely severe depression (Lovibond P.F. and Lovibond, 1995; Lovibond S.H. and Lovibond, 1995; Henry and Crawford, 2005). On average, the current sample had asymptomatic depression (T1: *M* = 8.50) but with considerable variability up to extremely severe depression (range = 0–40) at T1.

### MRI Acquisition

The MRI was collected on a 3.0T short bore GE Signa Excite system. High resolution T1 spoiled gradient recalled (SPGR) images were acquired in a sagittal orientation (TR = 8.2 ms; TE = 3.2 ms; FOV = 24 cm; flip angle = 12°; voxel size = 1 mm × 0.9375 mm × 0.9375 mm). Diffusion weighted images (DWI) were collected using an echoplanar pulse sequence with 70 contiguous 2 mm axial slices and 38 non-collinear diffusion gradients (TR = 10 s; TE = 77.99 ms; *b*-value = 800 s/mm^2^; FOV = 25.6 cm; flip angle = 90°; voxel size = 2 mm × 2 mm × 2 mm).

### Diffusion Tensor Imaging Image Processing

Anatomical T1 scans were processed using the *recon-all* pipeline in FreeSurfer v5.3 and reconstructions were visually inspected for quality control (Fischl, 2012). Diffusion weighted images (DWI) were preprocessed using a standardized pipeline in TRACULA (Yendiki et al., 2011). First, DWIs were corrected for image and eddy current distortions then registered to anatomical T1 scans using FreeSurfer’s *bbregister* affine transformation. T1 scans were then registered to a standard template (MNI152) for group comparison. Head motion (translation and rotation) was calculated within TRACULA and used as a nuisance covariate in subsequent analyses. For tract reconstruction, FSL’s *bedpost* method was used to fit the ball-and-stick model with two anisotropic compartments at every voxel to account for crossing fibers. TRACULA then conducts global probabilistic tractography to reconstruct the pathways of interest. An atlas of 33 healthy individuals’ manually reconstructed pathways is referenced for pathway reconstruction.

**FIGURE 1:**
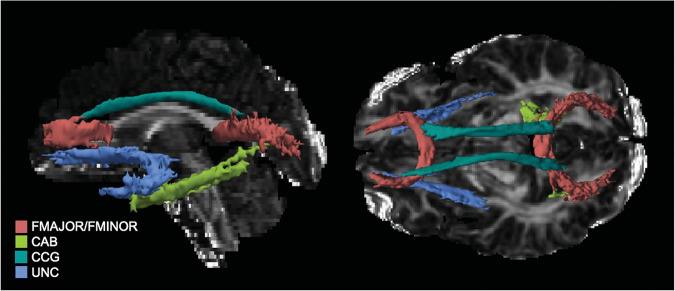
Tracts of interest as reconstructed in TRACULA for a representative participant. UNC, uncinate fasciculus; CCG, anterior cingulum; CAB, posterior, FMAJOR, forceps major; FMNIOR, forceps minor.

For the tracts of interest in the current study TRACULA segments the cingulum into anterior and posterior segments, and the CC into forceps major and minor. In all, 5 tracts—anterior cingulum (CCG), posterior cingulum (CAB), forceps major (FMAJOR), forceps minor (FMINOR), and uncinate (UNC)—were reconstructed and FA metrics extracted for analysis (see Figure 1 for tracts from a representative subject). Path reconstruction failed or was poor quality (i.e., fragmented tracts) for a handful of subjects in select tracts: left (*n* = 29) and right CAB (*n* = 44), and left (*n* = 11) and right UNC (*n* = 16). Thus, the smallest sample for any tract comparison was *n* = 104, as there was no further missing data for any of the covariates. For completeness, the remaining tracts constructed by TRACULA were also evaluated; however, since they were not of interest to the specific aims of the current study, results are presented in the Supplementary Material.

### Statistical Analysis

Fractional anisotropy values were extracted from TRACULA and used in separate general linear models (GLM) for each tract and hemisphere (8 GLMs: left and right uncinate, left and right anterior cingulum, left and right posterior cingulum, forceps major, and forceps minor) using R (R Core Team, 2020). A Benjamini–Hochberg correction was applied to correct for multiple comparisons (α = 0.05, Benjamini and Hochberg, 1995). For each regression, T2 PTSD symptom severity from the CAPS-5 was the outcome variable and T1 FA was the predictor while controlling for additional covariates. Head motion parameters were included as covariates along with sex (dummy coded: 0 = male, 1 = female) and age to account for potential sex and age differences that have been well documented in the literature (Hsu et al., 2008; Lebel and Beaulieu, 2011; Averill et al., 2018). Prior trauma history (weighted LEC total) was also included as a covariate.

Head motion parameters (average rotation and translation) were calculated within TRACULA. Pearson correlations were used to assess relationships between head motion and FA. Average translation at T1 was related to right (*r* = −0.21, *p* = 0.02) and left uncinate FA, *r* = −0.22, *p* = 0.01. In the current study, at T1, males had significantly greater FA than females in the right uncinate (*t* = 2.44, *p* = 0.01) and right anterior cingulum (*t* = 2.26, *p* = 0.02). Females had significantly greater FA in the left posterior cingulum (*t* = −2.00, *p* = 0.04). Bivariate correlations of T1 tract integrity and T1 PCL-5 scores were also examined. While there were no significant correlations between T1 FA and T1 PCL-5 total severity (all *p* > 0.05), T1 PCL-5 scores were included in the regression models to control for baseline PTSD symptoms at the time of the DTI scan.

Here is an example of the full GLM with all covariates for the right hemisphere UNC:

*CAPS (T2)* ∼ *RH_UNC_FA (T1)* + *head motion (T1)* + *age* + *sex* + *PCL-5 (T1)* + *LEC (T1)* + *DASS Dep (T1).*

## Results

### Bivariate Correlations

**FIGURE 2:**
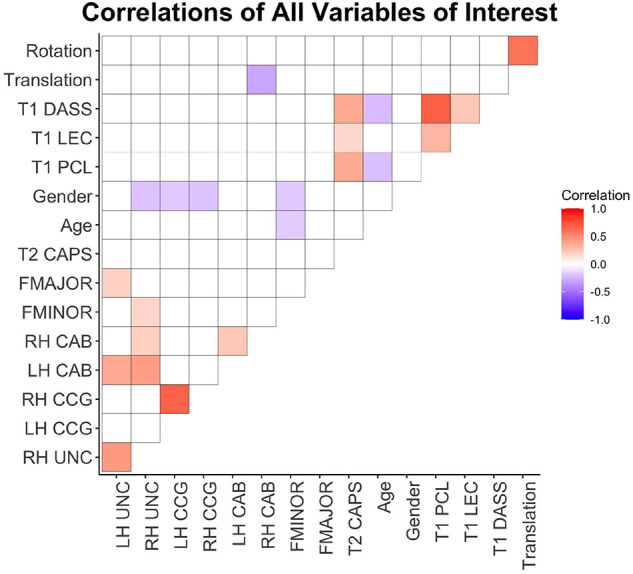
Correlation heatmap of pairwise Pearson correlations between all variables of interest. Warm colors indicate positive correlations and cool colors represent negative correlations. White cells represent non-significant pairwise correlations (*p* > 0.05). T1, 2-weeks post-injury; T2, 6-months post-injury; DASS, depression symptom subscale from DASS; LEC, weighted total LEC; PCL, PTSD Checklist; CAPS, Clinician Administered PTSD Scale; FMAJOR, forceps major; FMINOR, forceps minor; CAB, posterior cingulum; CCG, anterior cingulum; UNC, uncinate fasciculus; LH, left hemisphere; RH, right hemisphere.

See Figure 2 for a visual representation of all pairwise correlations for all variables of interest. T1 PTSD scores were significantly related with T2 PTSD (*r*^2^ = 0.13, *p* < 0.001). Lifetime trauma exposure was significantly related to T1 (*r*^2^ = 0.09, *p* < 0.001) and T2 PTSD (*r*^2^ = 0.02, *p* = 0.05), and T1 depression (*r*^2^ = 0.05, *p* < 0.01). Age was not significantly related to T2 PTSD (*p*’s > 0.05), and there were no sex differences in T1 or T2 PTSD or T1 depression (all *p*’s > 0.05). Age was negatively related to T1 depression (*r*^2^ = 0.04, *p* < 0.01). Depression and PCL scores at T1 were highly correlated (*r*^2^ = 0.47, *p* < 0.001), and T1 depression and T2 PTSD were moderately correlated (*r*^2^ = 0.13, *p* < 0.001).

Men had significantly higher FA than women in the right UNC [*t*(122) = 2.29, *p* = 0.02], right CCG [*t*(142) = 2.54, *p* = 0.01], left CCG [*t*(132) = 2.14, *p* = 0.03], FMINOR [*t*(132) = 2.15, *p* = 0.03]. Age was negatively related to FMINOR (*r*^2^ = 0.02, *p* = 0.04).

### Tract Analysis Results

Given the variability in time between index trauma and time of scanning (Table 1), bivariate correlations between T1 FA and days since injury at T1 scanning were evaluated. There were no significant relationships between T1 FA and days since injury at T1 scanning, and thus the variability in T1 FA cannot be attributed to variability in time since trauma.

With the high comorbidity of PTSD and depression in trauma samples, we ran the GLMs including the T1 DASS depression scores as a covariate, however, the results did not differ from if the DASS was excluded. This may stem from the high degree of correlation of depression and PCL-5 scores at T1 as well as moderate correlation of T1 depression and T2 CAPS. Therefore, to avoid multicollinearity and for parsimony, we present the results of the GLMs excluding T1 depression as a covariate below.

**FIGURE 3:**
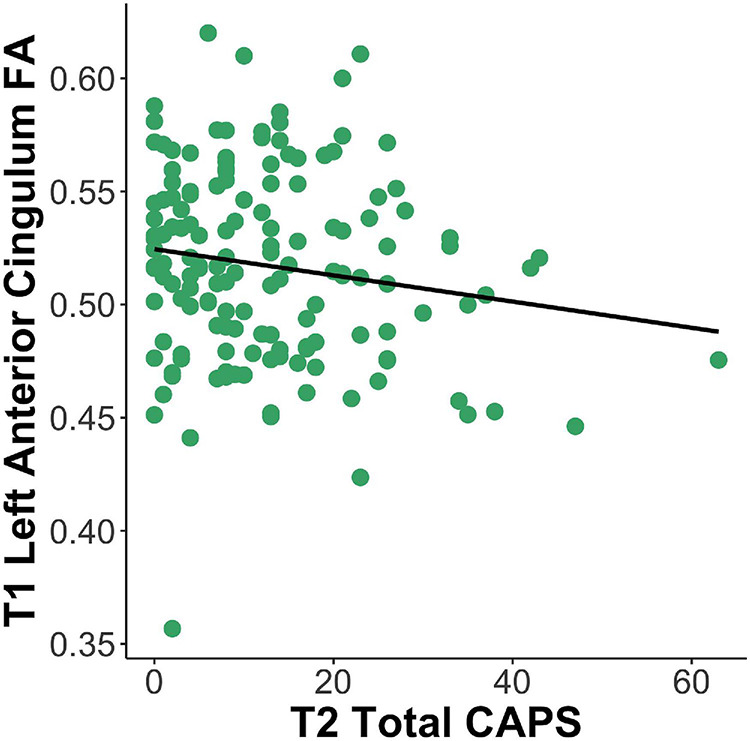
Lesser left anterior cingulum FA at T1 is related to greater total PTSD symptoms at T2 (β = –48.13, *p* = 0.003). FA, fractional anisotropy; CAPS, Clinician Administered PTSD Scale; T1, 2-weeks post-trauma; T2, 6-months post-trauma.

After correction for multiple comparisons, results of the tract-based analysis showed a marginal negative relationship between anterior cingulum FA at T1 and total CAPS-5 at T2 (adjusted *p* = 0.06), e.g., lower left anterior cingulum FA was related to greater total CAPS-5 symptoms (see Figure 3 and Table 2). No other tract results survived correction for multiple comparisons using the Benjamini–Hochberg method (all adjusted *p* > 0.09). Not surprisingly, in all models, T1 PCL-5 symptoms were significantly related to T2 CAPS-5. No other covariates (e.g., age, sex, head motion parameters, T1 LEC) significantly related to T2 CAPS-5.

**TABLE 2:** General linear models with all covariates (T1 DTI predicting T2 total CAPS symptoms).

		Left hemisphere			Right hemisphere		
		β	*CI*	*p*	β	***CI***	*p*
Uncinate fasciculus	Intercept	−11.31	−30.80 – 8.18	0.25	−4.31	−28.94 – 20.32	0.73
	FA	42.36	3.74 – 80.99	0.03^#^	19.75	−30.15 – 69.64	0.43
	Age	−0.08	−0.26 – 0.10	0.38	−0.07	−0.26 – 0.11	0.42
	Sex	0.64	−3.09 – 4.38	0.73	0.31	−3.55 – 4.16	0.87
	T1 PCL-5	0.24	**0.13 – 0.35**	<0.001*	**0.25**	**0.14 – 0.37**	<0.001*
	T1 LEC	0.05	−0.07 – 0.16	0.42	0.02	−0.10 – 0.14	0.75
	T1 translation	6.17	−0.17 – 12.50	0.05	5.22	−1.24 – 11.68	0.11
	T1 rotation	−1156.31	−2073.85 − –238.77	0.01^#^	−684.20	−1834.97 – 466.56	0.24
Anterior cingulum	Intercept	33.51	8.07 – 58.95	0.01	23.09	−1.27 – 47.45	0.06
	FA	−53.28	−94.69 −−11.87	0.01^#^	−35.88	−76.90 – 5.14	0.08
	Age	−0.06	−0.22 – 0.11	0.49	−0.06	−0.23 – 0.10	0.46
	Sex	−0.99	−4.52 – 2.54	0.58	−0.73	−4.31 – 2.85	0.68
	T1 PCL-5	**0.25**	**0.14 – 0.36**	<0.001*	**0.24**	**0.13 – 0.35**	<0.001*
	T1 LEC	0.01	−0.10 – 0.12	0.87	0.02	−0.09 – 0.13	0.65
	T1 translation	4.34	−1.63 – 10.32	0.15	4.93	−1.11 – 10.98	0.11
	T1 rotation	−771.95	−1607.28 – 63.37	0.07	−812.71	−1659.03 – 33.61	0.066
Posterior cingulum	Intercept	−5.57	−27.34 – 16.19	0.61	−3.32	−28.27 – 21.64	0.79
	FA	16.07	−31.67 – 63.81	0.51	16.37	−34.76 – 67.49	0.53
	Age	−0.01	−0.20 – 0.19	0.94	−0.01	−0.23 – 0.22	0.94
	Sex	0.01	−4.07 – 4.10	0.99	−1.41	−6.02 – 3.20	0.55
	T1 PCL-5	**0.24**	**0.12 – 0.37**	<0.001*	**0.26**	**0.13 – 0.39**	<0.001*
	T1 LEC	0.08	−0.05 – 0.20	0.22	0.04	−0.10 – 0.18	0.57
	T1 translation	5.55	−1.92 – 13.01	0.14	6.21	−1.35 – 13.77	0.11
	T1 rotation	−764.99	−1991.61 – 461.63	0.22	−904.55	−2214.89 – 405.79	0.17
Forceps minor	Intercept	8.99		−12.93 – 30.90		0.42	
	FA	−9.43		−49.51 – 30.66		0.64	
	Age	−0.03		−0.20 – 0.14		0.75	
	Sex	−0.64		−4.26 – 2.98		0.73	
	T1 PCL-5	**0.23**		**0.12 – 0.34**		<0.001*	
	T1 LEC	−0.00		−0.11 – 0.11		0.99	
	T1 translation	4.49		−1.72 – 10.71		0.15	
	T1 rotation	−760.61		−1618.91 – 97.69		0.08	
Forceps major	Intercept	−0.52		−28.45 – 27.42		0.97	
	FA	10.21		−31.06 – 51.49		0.62	
	Age	−0.08		−0.29 – 0.13		0.47	
	Sex	0.27		−3.98 – 4.51		0.90	
	T1 PCL-5	**0.21**		**0.08 – 0.33**		0.002*	
	T1 LEC	0.05		−0.08 – 0.19		0.44	
	T1 translation	6.07		−1.08 – 13.23		0.09	
	T1 rotation	−1063.73		−2030.91 – −96.55		0.03	

T1, 2-weeks post-trauma; T2, 6-months post-trauma; CAPS, Clinician Administered PTSD Scale; LEC, life events checklist total weighted score; FA, fractional anisotropy. *p-*values presented are uncorrected, bolded values with * indicates results that survived Benjamini–Hochberg correction (α = 0.05). ^#^ indicates marginal results after correction (*p* adjusted < 0.10).

As a follow-up, we also ran the GLM’s with head motion as the only covariate (excluding age, sex, and T1 PCL-5, T1 LEC), and the results were identical, indicating the results are robust with and without theoretically relevant covariates. Furthermore, we repeated the whole analysis with the PCL-5 at T2 as the outcome variable rather than CAPS-5, and still the results did not change, though this result was not surprising due to the high correlation of T2 PCL-5 and CAPS-5 in the current sample *r* = 0.51, *p* < 0.001.

## Discussion

The current study investigated the relationship of acute white matter integrity post-trauma and chronic PTSD symptoms. Based on previous literature, we expected a negative relationship between the cingulum, CC, and UNC integrity at T1 and overall PTSD symptom severity at T2. Our results partially support these hypotheses, in that lesser integrity of the anterior cingulum at T1 was marginally related to greater total PTSD symptoms at T2. However, there was no relationship between CC or UNC integrity at T1 and PTSD symptoms at T2.

The marginal results in the current study align with a large body of previous work implicating a negative relationship between integrity of the anterior cingulum and PTSD symptoms (Daniels et al., 2013; Jenkins et al., 2016; Siehl et al., 2018; Ju et al., 2020), and further show that integrity is related to chronic non-remitting PTSD symptoms following trauma exposure. The anterior cingulum runs the length of the cingulate gyrus connecting medial prefrontal cortices (mPFC) with the posterior cingulate cortex (PCC). The mPFC coordinates responsivity to salience in an adaptive way, based on previous experiences (Euston et al., 2012). The PCC is a critical structure in the default mode network, where it is implicated in internally-focused thought (Leech and Sharp, 2014). Therefore, lesser structural connectivity between the mPFC and PCC in the acute aftermath of trauma may indicate unregulated and maladaptive activity that over time may result in re-experiencing or intrusive PTSD symptoms (Siehl et al., 2018). Degradation of this tract may lead to a reduction in top-down control of the prefrontal cortices over the PCC, leaving the PCC unregulated and free to engage in activity that enables symptom development and prevents recovery.

Previous work has demonstrated the left and right cingulum have unique microstructural properties that can be detected using DTI (Jones et al., 2013). In addition, a slight bias toward changes within left hemisphere structure and function in relation to PTSD symptomology has been previously reported (Bremner, 2006). These observations lend support for the current finding that specifically left, but not right, cingulum integrity was marginally related to PTSD symptoms.

Though the anterior cingulum finding aligns with the literature, the relationship demonstrated in the current sample was not robust. Further, no other T1 tract integrity, even in the supplemental analysis, demonstrated a relationship with chronic PTSD symptoms. These results were unexpected, but we offer up a few explanations. First, only 18% of participants (13 male and 14 female) met criteria for PTSD diagnosis according to the CAPS-5 at T2. Thus, there may not be sufficient variability in PTSD symptom severity or this sample may not have severe enough symptoms (*M* = 12.70, *SD* = 11.25) to effectively see differences in white matter. Especially in the acute stages after traumatic injury white matter integrity differences may not be apparent so early after injury. Alternatively, white matter integrity may not be significantly related to subclinical PTSD.

Though our results trended toward *lesser* anterior cingulum related to *greater* PTSD symptom severity, many mixed findings of white matter integrity and symptoms in PTSD have been reported in the literature including other null results. These mixed findings could reflect how tract differences may differentially relate to symptoms based on whether you’re examining them as an acute post-trauma marker of risk or for chronic symptoms (when someone may have had symptoms for years). Most research of white matter integrity in PTSD has been done cross-sectionally, often *after* individuals have been diagnosed with PTSD, and compared across diagnostic groups rather than examining symptom severity as a continuous variable (Siehl et al., 2018; Dennis et al., 2019; Suo et al., 2019; Ju et al., 2020). The current study extends the literature by examining acute integrity of white matter pathways related to chronic PTSD symptom severity and subclinical symptoms, rather than PTSD diagnosis, a more clinically relevant approach to early treatment intervention targeting specific symptoms in trauma survivors.

It is important to note that patterns or changes in white matter integrity in the acute aftermath of trauma likely do not reflect structural brain changes that result directly from the index trauma. Rather, the integrity at this time point may reflect a general biological vulnerability or the preexisting accumulation of life stress and/or traumatic experiences prior to and in addition to the index trauma. It is near impossible to adequately separate prior trauma and stress from the index trauma or to effectively evaluate the relationship between the index trauma and its effect on the brain. Nonetheless, we believe evaluation of brain structure and function in the acute period after trauma still has predictive utility as it likely reflects biological vulnerability to the effects of trauma, possibly as a result of stress by providing a baseline measurement to then evaluate changes over time. To clarify these nuances, there is clear need to examine how changes in tract integrity post-trauma may provide better context of risk conference and symptom development through the use of longitudinal study designs with MRI scans at several time points post-trauma.

### Limitations

The current study is not without limitations. The requirement to score at least a three on the Predicting PTSD Questionnaire resulted in this study over-sampling individuals with high PTSD risk. Consequently, the results may not generalize to all traumatically injured samples. Similarly, mechanisms of injury varied across the sample. Various trauma types (i.e., assaultive or non-assaultive) may confer different likelihoods of trauma-related psychopathology (Conrad et al., 2017; Jakob et al., 2017); however, the sample was underpowered to adequately assess for these differences. In addition, the sample had moderate exposure to previous trauma, measured by the LEC, (*M* = 31.29, *SD* = 15.83) which has been shown to increase risk for PTSD and other psychopathology (Karam et al., 2014; Shalev et al., 2019) and may have confounded measurement of structural integrity and symptomology. Furthermore, self-reported symptomology (i.e., PCL-5) may be confounded by proximity to trauma, particularly at T1. Future research should continue to examine white matter as a predictor of psychopathology in the aftermath of trauma in a larger sample where variability in sample characteristics can be more appropriately accounted. Finally, owing to the sensitive population (e.g., recently injured trauma victims), we experienced loss of data due to head motion, likely a result of individual’s physical conditions making it difficult to lie still during MRI scanning.

### General Conclusion

In a traumatically injured sample with predominantly subclinical PTSD, the current study found no robust relationship between acute white matter integrity and chronic PTSD symptoms. These findings highlight the importance of timing when evaluating brain structure as a predictor of post-trauma outcomes. Despite the null findings, examination of brain structure and function in the acute post-trauma period is a critical for understanding risk of PTSD that may ultimately inform more effective treatments for trauma survivors.

## Data Availability Statement

The datasets presented in this article are not readily available because we currently do not have IRB approval to share these data, but are working with them to see our data sharing options. Requests to access the datasets should be directed to CW, cweis@mcw.edu.

## Ethics Statement

The studies involving human participants were reviewed and approved by Medical College of Wisconsin Institutional Review Board. The patients/participants provided their written informed consent to participate in this study.

## Author Contributions

CW, Td-C, and CL contributed to the conception and design of the study. CW organized and analyzed the data and wrote the initial draft. All authors contributed to manuscript revision, read, and approved the submitted work.

## Conflict of Interest

The authors declare that the research was conducted in the absence of any commercial or financial relationships that could be construed as a potential conflict of interest.

## Publisher’s Note

All claims expressed in this article are solely those of the authors and do not necessarily represent those of their affiliated organizations, or those of the publisher, the editors and the reviewers. Any product that may be evaluated in this article, or claim that may be made by its manufacturer, is not guaranteed or endorsed by the publisher.
