# Supplementary material for: Acute White Matter Integrity Post-trauma and Prospective Posttraumatic Stress Disorder Symptoms
Source: Front Hum Neurosci. 2021 Sep 29;15:742198. doi: 10.3389/fnhum.2021.742198 (PMC8511512; doi:10.3389/fnhum.2021.742198)
Supplement: Supplementary file 1 [file Data_Sheet_1.pdf]

Supplementary Material

Additional TRACULA tract analysis

For completeness, T1 FA from the remaining tracts constructed by TRACULA: anterior thalamic radiation (ATR), corticospinal tract (CST), inferior longitudinal fasciulus (ILF), parietal portion of superior longitudinal fasciulus (SLFP), and temporal portion of superior longitudinal fasciulus (SLFT) were evaluated in the same way as described in the main text. Path reconstruction failed or was poor quality (i.e., fragmented tracts) for a handful of subjects in select tracts: FMINOR (n = 13), FMAJOR (n = 34), left (n = 1) and right CST (n = 1), left (n = 29) and right CAB (n = 44), left (n = 1) and right ILF (n = 3), and left (n = 11) and right UNC (n = 16). Thus, the smallest sample for any tract comparison was n = 104, as there was no further missing data for any of the covariates.

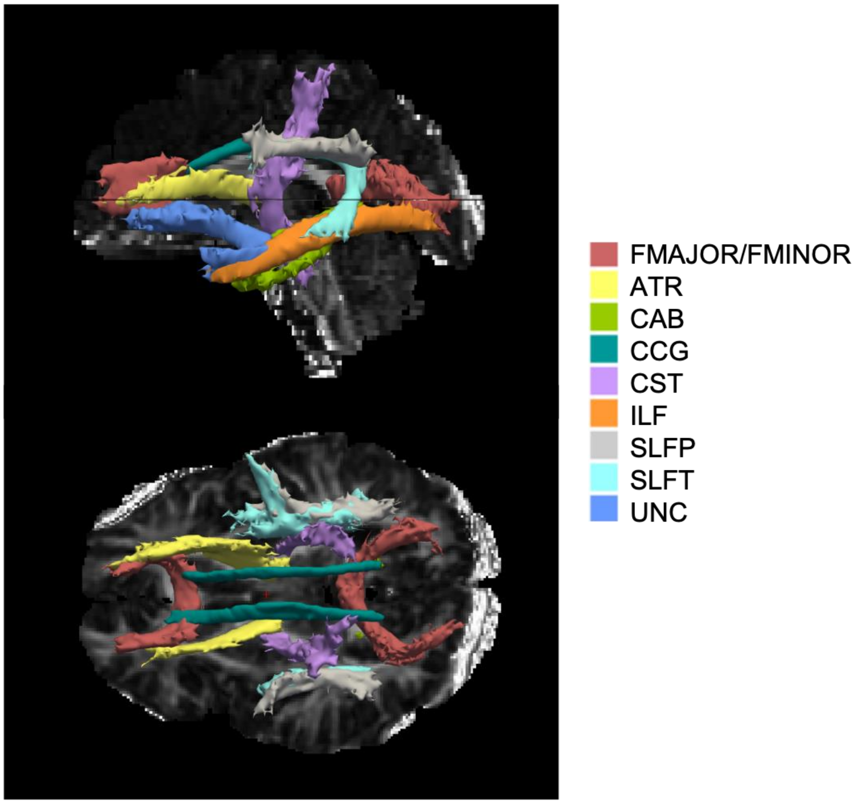

Supplementary Figure 1. Tracts of interest as reconstructed in TRACULA for a representative participant. **FMAJOR,** forceps major (red); **FMINOR,** forceps minor (red); **ATR**, anterior thalamic radiation (yellow); **CAB**, posterior cingulum (lime green); **CCG**, anterior cingulum (teal); **CST**, corticospinal tract (lavender); **ILF**, inferior longitudinal fasciculus (orange); **SLFP**, superior longitudinal fasciculus parietal segment (grey); **SLFT**, superior longitudinal fasciculus temporal segment (light blue); **UNC**, uncinate fasciculus (dark blue).

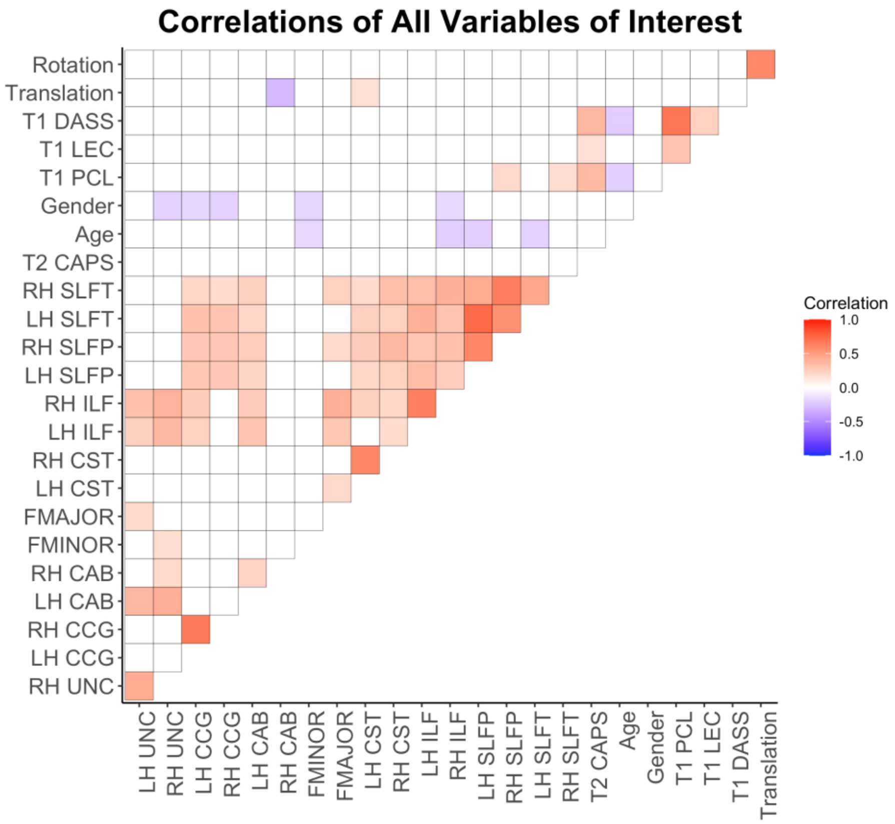

Supplementary Figure 2. Correlation heatmap of pairwise Pearson correlations between all variables of interest. Warm colors indicate positive correlations and cool colors represent negative correlations. White cells represent non-significant pairwise correlations ($p > 0.05$). **T1,** 2-weeks post-injury; **T2,** 6-months post-injury; **DASS,** depression symptom subscale from DASS; **LEC,** weighted total LEC; **PCL**, PTSD Checklist; **CAPS**, Clinician Administered PTSD Scale; **SLFT**, superior longitudinal fasciculus temporal portion; **SLFP**, superior longitudinal fasciculus parietal portion; **ILF**, inferior longitudinal fasciculus; **CST,** corticospinal tract; **FMAJOR**, forceps major; **FMINOR**, forceps minor; **CAB**, posterior cingulum; **CCG**, anterior cingulum; **UNC**, uncinate fasciculus.

FA values were extracted from TRACULA and used in separate general linear models (GLM) for each tract and hemisphere. For each regression, T2 PTSD symptom severity from the CAPS-5 was the outcome variable and T1 FA was the predictor while controlling for additional covariates. Head motion parameters were included as covariates along with sex, age, T1 PTSD,

and T1 LEC scores. A Benjamini-Hochberg correction was applied to correct for multiple comparisons (α=0.05, Benjamini & Hochberg, 1995).

Here is an example of the full GLM with all covariates for the right hemisphere UNC:

CAPS (T2) ~ RH_UNC_FA (T1) + head motion (T1) + age + sex + PCL-5 (T1) + LEC (T1)

Men had significantly higher FA than women in the right ATR, $t(117) = 2.21$, $p = 0.02$. Age was negatively related to right ATR ($r^2 = 0.03$, $p = 0.02$) and ILF ($r^2 = 0.04$, $p = 0.01$), and left SLFP ($r^2 = 0.04$, $p = 0.01$) and SLFT ($r^2 = 0.03$, $p = 0.01$).

After correction for multiple comparisons, results of the supplemental tract-based analysis showed no relationship between tract FA at T1 and total CAPS-5 at T2 (Supplemental Table 1). The addition of DASS depression scores from T1 as a covariate again did not change the results; still no tract FA at T1 was related to T2 PTSD.

Supplemental Table 1 General Linear Models with all covariates (T1 DTI predicting T2 Total CAPS symptoms)

		Left Hemisphere			Right Hemisphere		
		β	*CI*	*p*	β	*CI*	*p*
Anterior Thalamic Radiation	Intercept	27.77	1.17 – 54.37	0.04	29.31	1.80 – 56.81	0.03
	FA	-59.20	-119.74 – 1.35	0.05	-59.74	-119.50 – 0.02	0.05
	Age	-0.06	-0.22 – 0.11	0.51	-0.08	-0.24 – 0.09	0.38
	Sex	-0.60	-4.13 – 2.93	0.74	-0.81	-4.38 – 2.75	0.65
	T1 PCL-5	**0.23**	**0.12 – 0.34**	**<0.001***	**0.23**	**0.14 – 0.35**	**<0.001***
	T1 LEC	0.05	-0.06 – 0.16	0.40	0.04	-0.07 – 0.15	0.43
	T1 Translation	5.38	-0.69 – 11.45	0.08	5.67	-0.44 – 11.78	0.07
	T1 Rotation	-760.91	-1604.14 – 82.32	0.07	-887.76	-1737.80 – 37.72	0.04
Corticospinal Tract	Intercept	29.67	-6.86 – 66.19	0.11	36.89	3.31 -70.48	0.03
	FA	-62.06	-145.37 – 21.26	0.14	-79.99	-157.54 - -2.43	0.04
	Age	-0.06	-0.23 – 0.11	0.47	-0.05	-0.22 – 0.12	0.56
	Sex	0.04	-3.47 – 3.56	0.98	-0.35	-3.87 – 3.16	0.84
	T1 PCL-5	**0.23**	**0.12 – 0.34**	**<0.001***	**0.24**	**0.13 – 0.35**	**<0.001***
	T1 LEC	0.04	-0.07 – 0.15	0.48	0.04	-0.07 – 0.15	0.48
	T1 Translation	5.68	-0.49 – 11.86	0.07	5.64	-0.47 – 11.75	0.07
	T1 Rotation	-817.77	-1667.61 – 32.07	0.06	-846.14	-1693.92 – 1.64	0.05
Inferior Longitudinal Fasciculus	Intercept	3.53	-23.75 – 30.80	0.80	15.17	-10.25 – 40.59	0.24
	FA	1.63	-51.05 – 54.32	0.95	-20.65	-65.77 – 24.47	0.37
	Age	-0.05	-0.22 – 0.12	0.56	-0.07	-0.24 – 0.11	0.45
	Sex	0.06	-3.48 – 3.60	0.97	-0.49	-4.12 – 3.14	0.79
	T1 PCL-5	**0.23**	**0.12 – 0.34**	**<0.001***	**0.24**	**0.13 – 0.35**	**<0.001***
	T1 LEC	0.04	-0.07 – 0.15	0.51	0.03	-0.08 – 0.14	0.59
	T1 Translation	4.76	-1.34 – 10.87	0.12	4.58	-1.54 – 10.70	0.14
	T1 Rotation	-797.81	-1655.54 – 59.93	0.07	-805.87	-1665.38 – 53.64	0.06
Superior Longitudinal Fasciculus (Parietal)	Intercept	18.72	-9.03 – 46.48	0.18	18.21	-10.07 – 46.48	0.20
	FA	-34.38	-94.92 – 26.17	0.26	-33.16	-95.14 – 28.82	0.29
	Age	-0.07	-0.24 – 0.10	0.41	-0.06	-0.23 – 0.11	0.50
	Sex	0.25	-3.30 – 3.80	0.89	-0.08	-3.59 – 3.44	0.96
	T1 PCL-5	**0.22**	**0.12 – 0.33**	**<0.001***	**0.23**	**0.13 – 0.34**	**<0.001***
	T1 LEC	0.04	-0.07 – 0.15	0.49	0.04	-0.07 – 0.15	0.50
	T1 Translation	4.35	-1.75 – 10.45	0.16	4.41	-1.69 – 10.50	0.15
	T1 Rotation	-710.82	-1568.77 – 147.13	0.10	-737.06	-1590.85 – 116.74	0.09
Superior Longitudinal Fasciculus (Temporal)	Intercept	11.11	-21.83 – 44.05	0.51	8.57	-18.14 – 35.27	0.53
	FA	-15.77	-89.10 – 57.56	0.67	-10.38	-71.51 – 50.74	0.74
	Age	-0.06	-0.23 – 0.11	0.50	-0.05	-0.22 – 0.12	0.55
	Sex	0.05	-3.50 – 3.60	0.97	-0.05	-3.57 – 3.48	0.97
	T1 PCL-5	**0.23**	**0.12 – 0.34**	**<0.001***	**0.23**	**0.12 – 0.34**	**<0.001***
	T1 LEC	0.04	-0.07 – 0.15	0.51	0.04	-0.07 – 0.15	0.51
	T1 Translation	4.58	-1.53 – 10.68	0.14	4.63	-1.47 – 10.73	0.13
	T1 Rotation	-756.67	-1614.74 – 101.39	0.08	-773.96	-1627.76 – 79.84	0.07

T1, 2-weeks post-trauma; T2, 6-months post-trauma; CAPS, Clinician Administered PTSD Scale; FA, fractional anisotropy. *p* values presented are uncorrected, * indicates results that survived Benjamini-Hochberg correction (α=0.05).
